# Research Progress on the Preparation and Action Mechanism of Natural Deep Eutectic Solvents and Their Application in Food

**DOI:** 10.3390/foods11213528

**Published:** 2022-11-05

**Authors:** Kairong Wu, Jing Ren, Qian Wang, Maheshati Nuerjiang, Xiufang Xia, Chun Bian

**Affiliations:** 1 College of Food Science, Northeast Agricultural University, Harbin 150030, China; 2 School of Food Engineering, Harbin University, Harbin 150036, China

**Keywords:** natural deep eutectic solvent, green solvent, properties, mechanism, application

## Abstract

Natural deep eutectic solvent (NADES) is the eutectic mixture which is formed by hydrogen bond donors (HBDs) and hydrogen bond acceptors (HBAs) with a certain molar ratio through hydrogen bonding. NADES is a liquid with low cost, easy preparation, biodegradability, sustainability and environmental friendliness at room temperature. At present, it is widely used in food, medicine and other areas. First, the composition, preparation and properties of NADES are outlined. Second, the potential mechanism of NADES in freezing preservation, the removal of heavy metals from food and the extraction of phenolic compounds, and its application in cryopreservation, food analysis and food component extraction, and as a food taste enhancer and food film, are summarized. Lastly, the potential and challenges of its application in the food field are reviewed. This review could provide a theoretical basis for the wide application of NADES in food processing and production.

## 1. Introduction

The term natural deep eutectic solvent (NADES), originally proposed by Choi et al. [1], is a new class of mixtures formed by two or more liquids or solids in a certain ratio. The eutectic point of NADES is significantly lower than that of the individual components due to the formation of hydrogen bonds between the constituents [2]. In appearance, it appears as a thick, transparent liquid [3]. NADES is found in some cold-tolerant animal species such as frogs, insects and worms. It is mainly composed of biological metabolites such as sugars, amino acids, organic acids and choline derivatives, and usually also contains a certain mole ratio of water [1]. Structurally, NADES is composed of hydrogen bond donors (HBDs) and hydrogen bond acceptors (HBAs) through hydrogen bonding with a certain molar ratio. Therefore, the type of HBDs and HBAs, as well as the position and quantity of hydrogen bonds, have an effect on the stability of NADES [4].

NADES has the advantages of low production cost [5], simple synthesis [5,6], wide range of polarities [7] and biodegradability [8], and being sustainable, it can replace traditional solvents, especially because of its low or no toxicity [7,9]. Another important feature is that it usually possesses some noteworthy chemical characteristics, including low vapor pressure, relatively wide liquid range, good thermal stability or non-flammability. In addition, NADES is non-toxic, high-purity, and free of waste, so it can be directly added to food formulations as a new type of green solvent, which is its main advantage over traditional green solvents [10]. At present, studies on NADES are concerned with using it to extract various compounds, optimizing the extraction procedure and enhancing the stability of the extracts. There are few review articles on the mechanism of action of NADES and its application in food.

In this review, the composition, preparation method and characteristics of NADES are introduced, the potential mechanism of NADES is disclosed, and the research progress of NADES application in food is also summarized. This review could provide a theoretical reference for the widespread application of NADES in the food business.

## 2. Natural Deep Eutectic Solvents

### 2.1. Composition

**Figure 1:**
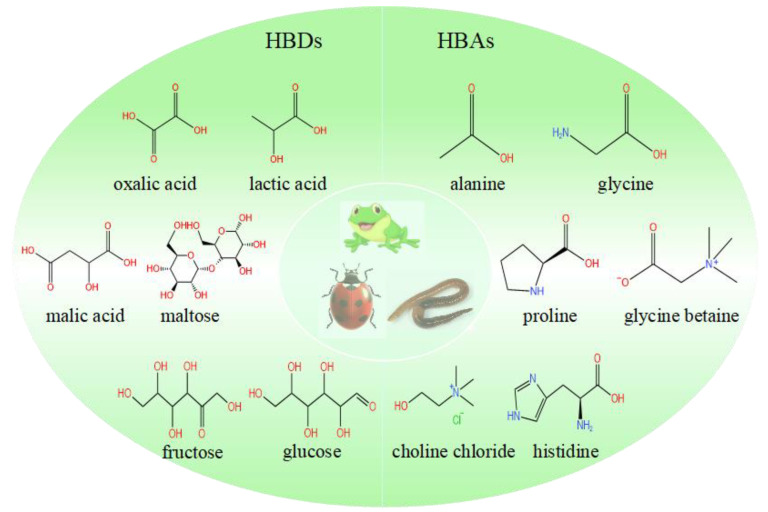
Chemical structures of common HBDs and HBAs.

Natural deep eutectic solvent (NADES) is composed of a variety of hydrogen bond donors (HBDs) and hydrogen bond acceptors (HBAs). Typical HBAs include nontoxic quaternary ammonium salts (e. g. choline chloride) and amino acids (e. g. alanine, glycine, proline, histidine, glycine betaine), while HBDs mainly include organic acids (e. g. oxalic acid, lactic acid, malic acid) and carbohydrates (e. g. maltose, fructose, glucose) (Figure 1). Alcohols, amines, aldehydes, ketones and carboxyl groups have dual properties, they can act as either HBA or HBD [5,8]. At present, 135 binary NADESs based on primary metabolites (PRIM) have been discovered [11], which can further form multi-component eutectic solvents, for instance, quaternary or ternary NADESs.

Other than PRIM, NADES can also be composed of more complicated highly evolutionary metabolites (HEVO) or natural products. There are three ways of formation [11]. The first is where NADES is composed of low melting point components such as menthone, D-limonene, p-cymene and menthol. The second is a NADES based on HEVO. For instance, the NADES consists of menthol and organic acids. The third is a NADES which contains glycoside natural substances. In addition, the other essential element of NADES is water, and the NADES prepared in most studies contains a specific molar ratio of water.

### 2.2. Preparation

**Figure 2:**
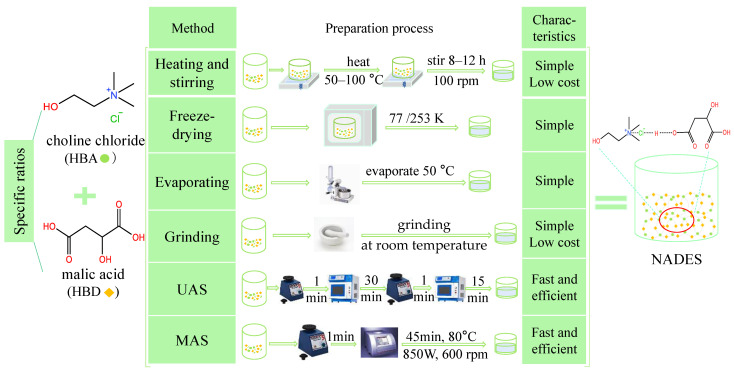
The preparation of NADES using choline chloride and malic acid by different methods [6,7,12,13,14,15].

For preparation, NADES is usually synthesized by six physical methods: heating and stirring, freeze-drying, evaporation, grinding, and ultrasound-assisted and microwave-assisted synthesis (Figure 2).

#### 2.2.1. Heating and Stirring

Accurately weigh each component and transfer it to an aluminum foil sealed beaker. Thereafter, the mixture is warmed to a certain temperature (50–100 °C) and stirred for approximately 8–12 h at 100 rpm with a magnetic stirrer equipped with a heating plate, until a kind of viscous and transparent liquid is formed [6,12].

#### 2.2.2. Freeze-Drying

Weigh each component accurately and add a specific molar ratio of water. Thereafter, the mixture is freeze-dried at 77 K and 253 K, and the water is sublimated to obtain NADES in its pure state [13].

#### 2.2.3. Evaporating

Accurately weigh each component and dissolve them in water, and they are subsequently placed on the rotary evaporator at 50 °C for evaporation. Finally, the solvent is placed in the desiccator until constant weight [7].

#### 2.2.4. Grinding

The mixture of the components is ground in a mortar and pestled at room temperature until a homogeneous liquid is formed [14].

#### 2.2.5. Ultrasound-Assisted Synthesis (UAS)

The accurately weighed mixture of each component is homogenized in a vortex for about 1 min, followed by treatment for 30 min in an ultrasonic bath. The above homogenization steps are repeated, followed by further treatment using an ultrasonic bath for 15 min [15]. After synthesis, NADES is stored in a desiccator at room temperature.

#### 2.2.6. Microwave-Assisted Synthesis (MAS)

The precisely weighed mixture of each component is homogenized in a vortex for around 1 min, followed by treatment for 45 min at 80 °C in a microwave reactor operated at 850 W and 600 rpm [15].

Among these methods, freeze-drying, evaporation and grinding are easy to operate, and ultrasonic and microwave-assisted synthesis are fast and efficient, because microwave radiation will interact with materials and cause dipole rotation, resulting in collisions between molecules and HBD and HBA components, and finally lead to dielectric heating, thus shortening the synthesis time [16]. Similarly, the cavitation effect caused by ultrasonic waves contributes to the interaction between HBD and HBA components [15]. However, the most common methods for preparing a NADES is heating and stirring, due to the fact that it is not only inexpensive and easy to operate, but also makes it simpler to regulate and change the manufacturing conditions during NADES synthesis, which is important when using thermally unstable components [17]. Santana et al. [15] used organic acid and xylitol as raw materials to synthesize NADES by three different methods, and compared their physical and chemical properties, demonstrating that NADESs obtained by different synthesis methods have similar properties.

### 2.3. Properties and Impact Factors

**Figure 3:**
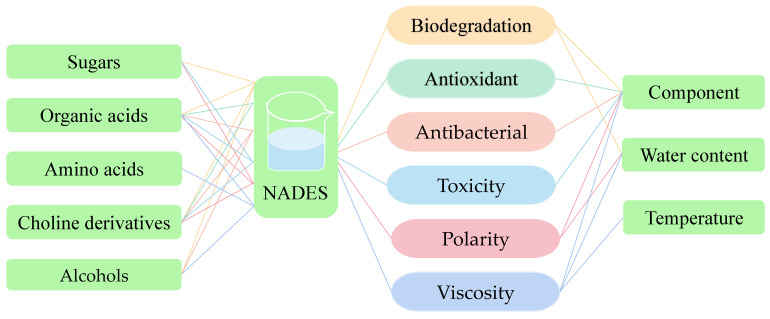
Properties and impact factors of NADES.

NADES has the characteristics of biodegradation, antioxidation, being antibacterial, low or no toxicity, wide polarity range and high viscosity, which are mainly affected by the components, water content and temperature (Figure 3).

#### 2.3.1. Properties

The biodegradation of substances is critical for the ecological environment and human health, and most of the reported NADESs are classified as compounds prone to biodegradation. Radošević et al. [18] investigated the degradation rate of NADESs composed of choline chloride, and the tests showed that the prepared NADESs were more than 60% biodegradable, thus they were called biodegradable. However, Wen et al. [19] found that only two of the eight NADESs tested were biodegradable.

Antioxidant activity improves the stability and shelf life of foods. Mitar et al. [20] found that organic acid-based NADESs had superior antioxidant activity through oxygen free radical measurement. However, a NADES which was composed of choline chloride and polyols showed low antioxidant activity. Therefore, a NADES can be prepared by using amino acids and organic acids with strong antioxidant properties as HBA and HBD to improve its antioxidant properties [21].

The antibacterial activity of NADESs can effectively preserve food from microbial contamination. Wen et al. [19] compared the growth status of *Escherichia coli* in media with and without NADES, and observed that NADES containing quaternary ammonium salt showed significant antimicrobial activity at higher concentrations. Similarly, Radošević et al. [18] explored how three bacteria including *Escherichia coli* responded to various NADESs, and discovered that a NADES which contained organic acids had strong antibacterial action, but its constituent parts did not show this property. Furthermore, NADES has a stronger inhibitory effect on Gram-negative bacteria than on Gram-positive bacteria, which is most likely a result of the components of the NADES interacting through hydrogen bonding with the polysaccharide or peptide chains of the cell wall, causing cell damage [22].

The toxicity of NADES determines its application in food. Radošević et al. [18] evaluated the toxicity of NADES for fish, wheat and human cell lines, and their data showed that three choline chloride-based NADESs had low cytotoxicity, but did not inhibit wheat seed germination. Paiva et al. [8] tested cell viability after exposure to different NADESs, and found that NADESs containing tartaric acid inhibited cell metabolic activity. However, Mitar et al. [20], regarding the toxicity tests of NADES on three human cell lines, revealed that even at the largest concentration (2000 mg L^−1^), NADES did not inhibit the growth of human HEK-293T, HeLa and McF-7 cells.

Furthermore, a key characteristic of NADES is its polarity, which has an impact on its solubility. The polarity range of NADES is broad, ranging from 44.81 kcal mol^−1^ to 50.07 kcal mol^−1^. Among them, NADESs composed of malic acid, choline chloride and water with a molar ratio of 1:1:2 had the largest polarity, followed by NADESs composed of fructose, glucose, sucrose and water, while NADESs prepared from sugar and polyols has the lowest polarity [7].

However, NADES has some limitations. The most obvious issue is the high viscosity, which can be as high as 720 mm^2^/s at 40 °C [7], and high viscosity can affect the flow of substances and reduce the extraction efficiency of NADES [23], so it needs to reduce viscosity to promote its application. The following two methods can be used to lessen NADES’s viscosity. The first is heating. Because of the increased intermolecular force at high temperatures and the structural damage brought on by thermal expansion, NADES viscosity reduces as temperature rises [24]. The second is water dilution, which causes a significant reduction in NADES viscosity as the hydrogen bonding connection between the components gradually weakens as the water content rises [6].

#### 2.3.2. Impact Factors

##### Components

The properties of NADES are primarily determined by its composition. Zhao et al. [22] measured the biodegradation values of 20 different NADESs. Among them, NADES composed of quaternary ammonium salt and urea had the highest biodegradability (97.1%), while NADES composed of quaternary ammonium salt and triethylene glycol exhibits the lowest biodegradability (69.3%). This is because choline chloride is easily biodegradable, reaching 93% degradation in 14 days. Similarly, Radoevi et al. [18] investigated the biodegradability of NADES with choline chloride as the HBA and discovered that the highest and lowest biodegradation levels were 96% and 68%, respectively, when the HBD was glycerol and oxalic acid.

Studies on the antioxidative and antibacterial properties of NADES have shown that organic acid-based NADESs exhibited better antibacterial activity and antioxidative properties compared with NADESs of other components [18,20]. The toxicity of NADES is also affected by their composition, because the decalcified charge in NADES damages the bacterial cell wall. Jesus et al. [25] found that the NADES made of betaine, glycerol, sucrose and water had higher toxicity than betaine, glycerol, trehalose and water at the same proportion (2:1:3:5). Furthermore, many studies have shown that NADES containing organic acids (e. g. malonic acid and tartaric acid) exhibits higher cytotoxicity in vitro than sugar-based NADES [7].

The components also affect the polarity and viscosity of NADES. According to the research data of Craveiro et al. [5], compared to NADESs made from choline chloride and sugar, those made from organic acids exhibit higher polarity; the higher the proportion of organic acids, the more polar the NADES is. In terms of the influence on viscosity, according to Altamash et al. [26], lactic acid was used to prepare NADES, which had good fluidity and low viscosity, as opposed to malic acid, which was used to prepare NADES with poor fluidity and high viscosity. Zannou et al. [27] showed that the viscosity of NADESs ranged from 0.39 to 2063.67 mPa, among which choline chloride–sugar and organic-sugar NADESs had the highest viscosity, while choline chloride–organic acid and choline chloride–alcohol NADESs had the lowest viscosity, which was consistent with the results obtained by Aroso et al. [28]. In addition, Xin et al. [29] discovered that the greater the percentage of choline chloride, the more difficult the liquid flow, that is, the higher the viscosity of NADES.

##### Water Content

Water content is another important factor affecting NADES characteristics. This is because the components of NADES form an extensive hydrogen bonding system which will gradually weaken with water dilution and will disappear when water content exceeds 50%. Water dilution is beneficial for the biodegradation of NADES. Studies have found that the degradation rate of undiluted NADES is slow, but if enough water is added to NADES before treatment, the rate of degradation is greatly improved [17]. The presence of water also increases the polarity of the NADES. Craveiro et al. showed that when water content increased by 4%, its polarity correspondingly increased by 1.2% [5]. Furthermore, viscosity decreases with increasing water content. After adding 5% water, NADES’s viscosity reduces by 1/3, and with 10% water, the viscosity reduces to 1/10 of the original value [7].

##### Temperature

The temperature mainly affects the viscosity of NADES. When the temperature increases from 313 K to 344 K, the viscosity of NADES consisting of L-isoleucine and tetrabutyl ammonium chloride is reduced from 1.58 Pa·s to 0.2 Pa·s [23]. Similarly, Dai et al. [7] investigated the viscosity change of NADES prepared with glucose, choline chloride and water (2:5:5) in the temperature range of 20 to 60 °C. The results showed that the viscosity decreased to 1/3 of its original value when the temperature increased to twice its original value. In addition, Aroso et al. [28] found that the viscosity of NADES consisting of choline chloride and xylose (1:1) is reduced from 100 Pa·s to 0.5 Pa·s when the temperature increased from 300 to 360 T.

## 3. Action Mechanism of Natural Deep Eutectic Solvent

### 3.1. Potential Mechanism of Natural Deep Eutectic Solvent as a Cryoprotectant

**Figure 4:**
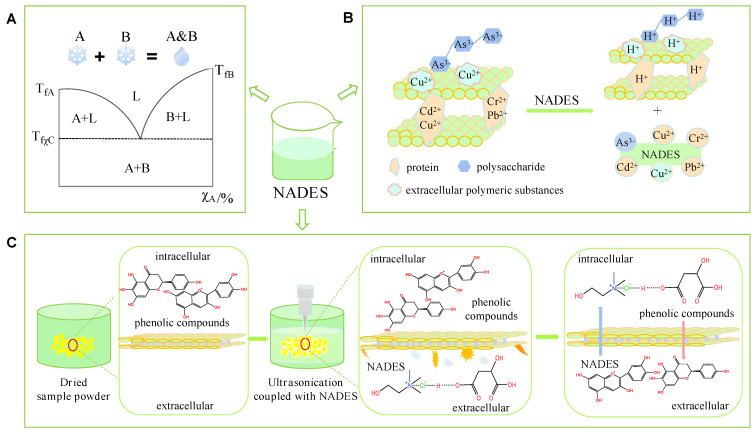
Mechanism of action of NADES. (**A**) Schematic phase diagram of the FP of NADES system [8]. χ_A_: the molar ratio of A to B (%); L: the mixture of A and B (liquid/liquid); A+B: the mixture of A and B (solid/solid); A+L: the mixture of A and B (liquid/solid or solid/liquid); B+L: the mixture of A and B (liquid/solid or solid/liquid); (**B**) removal of heavy metals from food by NADES [34,35]; (**C**) phenolic compounds were extracted by ultrasound-assisted NADES [36].

In Figure 4A, A and B are the components of NADES, and T_fχC_, T_fA_ and T_fB_ represent the freezing points (FP) of NADES, A and B, respectively, compared with the FP of a single component, the FP of NADES is strongly inhibited [8,11]. This is due to the fact that NADES is formed by the combination of HBAs and HBDs through hydrogen bonds. The charge delocalization effect in the hydrogen bond network system will lead to the FP decrease in NADES [30]. Numerous hydrogen bonds in the system provide supramolecular network structure for NADES, which significantly increases the viscosity of NADES [6]. Therefore, it is difficult to realize the molecular motion and reorientation of free water in the NADES system, which makes it hard for water to nucleate.

For ice nucleation, the two necessary conditions are the primary ice core and the subcooled water required for the ice growth process [31]. As the temperature decreases during freezing, the transverse relaxation time migration of NADES is obvious, demonstrating that at lower temperatures, the stability of the hydrogen bond network can be significantly increased, and its viscosity further increases, and water molecules bind more tightly [32]. A stronger hydrogen bond network can immobilize free water or hinder water reorientation, thus preventing supercooled water from entering the ice surface and making NADES accumulate more supercooled water without nucleation [33]. In general, the possible mechanism of NADES anti-freezing is that NADES inhibits molecular movement such as water movement in the system by enhancing the strength of hydrogen bond networks at low temperature, which makes it difficult for water molecules to nucleate or move to the ice core, thus inhibiting the nucleation and growth of ice crystals and providing the system with excellent anti-freezing ability.

### 3.2. Potential Mechanism of Heavy Metal Removal by Natural Deep Eutectic Solvent

Heavy metal removal mechanisms mainly include adsorption, ion exchange and precipitation. The method of heavy metal removal by NADES is adsorption, primarily chemisorption. As seen in Figure 4B, heavy metal ions act as HBAs to combine with organic compounds (HBDs) in food, primarily proteins, by hydrogen bond complexation. When acidic NADES are added to food contaminated with heavy metals, the introduced H^+^ will attack the electronic sites in the food and compete with metal ions for electron pairs, thus releasing them from the surface of the food and combining with NADES anions in the washing solution. That is, NADES removes heavy metals from food by destroying hydrogen bonds between metal ions and organic compounds [37].

The removal process is separated into two phases. The first phase is the quick removal phase, which is mainly controlled by the diffusion of the external surface. At this stage, the abundance of heavy metal ions on the food surface is relatively high, and NADES is able to provide numerous active adsorption sites that can be complexed with metal ions on the cell membrane, resulting in a significant increase in removal rate. The second phase is the slow removal phase, which is mostly the internal diffusion control stage. As the adsorption process progresses, NADES gradually enters the interior of the food for adsorption. However, because the number of active adsorption sites on its surface is gradually declining, the adsorption rate gradually slows down, and the adsorption process ends when NADES reaches saturation [34,35].

### 3.3. Potential Mechanism of Extracting Phenolic Compounds by Natural Deep Eutectic Solvent

The mechanism of phenolic compound extraction by NADES was revealed using pulsed ultrasonic-assisted extraction as an example. According to Figure 4C, the cell structure of the dry specimen is complete. When extraction is performed with ultrasound-assisted NADES, the intracellular structure is exposed due to the strong penetration and erosion ability of NADES into the cell wall. The more the intracellular structure is exposed, the more asymmetric collapsing jets will be generated, so that numerous NADESs will enter the cell interior. At the same time, intracellular components such as phenolic compounds flow out. Additionally, ultrasonic propagation mediated by NADES will undergo compression and rarefied cycles, and cavitation bubbles will be generated when the threshold is exceeded. The implosion and collapse of cavitation bubbles will affect the properties of the solvent and improve its extraction effect [36].

The microflow caused by the implosion and the micro-whirling generated by the nonlinear oscillation of the bubble can effectively mix the suspended particles with the solvent, break the barrier of the stagnant layer around the particles, and promote the mass transfer of intracellular components to the bulk solvent. The collapse mainly comes in two ways. On the one hand, the loosening and destruction of the food matrix will induce transient high shear force and local turbulence around the bubble, which is conducive to extraction. On the other hand, asymmetric collapse near the sample particle surface will also release a high-speed jet to the particle. The associated effects caused by the cavitation bubble will further damage the cell structure, causing more intracellular components to flow outward and obtain more target compounds. In addition, the low vapor pressure characteristic of NADES is conducive to the formation of high-strength cavitation, which greatly improves the extraction efficiency [38].

## 4. Applications of Natural Deep Eutectic Solvent

The applications of NADES in food mainly include five aspects: food cryoprotectant, determination and removal of heavy metals and other contaminants in food, extractant, food taste enhancers and food films (Table 1).

### 4.1. Cryoprotectant

Adverse reactions such as protein denaturation and lipid oxidation will occur during the frozen storage of meat products [52,53,54,55]. Cryoprotectants prevent protein denaturation during freezing and prolong the shelf life of meat products. NADES has the characteristics of changing the thermal behavior of water, inhibiting crystallization and leading to glass transition of water, so it has high potential as a cryoprotectant [12]. Lactic acid bacteria (LAB) is the most typical probiotic, which is frequently utilized in all types of food production. NADES is effective in penetrating LAB cells, inhibiting ice crystal formation and protein aggregation, and maintaining the activity of two intracellular enzymes. Therefore, NADES can significantly improve the survival of lactic acid bacteria during frozen storage [56]. NADES can also be used as antifreeze for food contact interfaces. The freezing resistance of NADES was reflected by the anti-frosting ability and deicing ability of NADES-coated steel substrate under extreme conditions. The results show that NADES has great potential as a green and safe antifreeze in the frozen food industry [32]. Moreover, Castro et al. [12] also proved that NADES showed excellent low-temperature protection ability in a non-toxic, economical and green way. It is noteworthy that NADESs used as cryoprotectants in different studies contained proline, which is probably because proline itself is an antifreeze agent with the advantages of safety, non-toxicity, wide source and low price.

### 4.2. Food Analysis

NADES has been used for the determination and removal of heavy metals, pesticide residues and other contaminants in food due to its non-toxic, biodegradable, green and sustainable characteristics. Cadmium rice is a global food safety issue. The cadmium removal effect of NADES on cadmium-contaminated rice flour showed that all the prepared NADESs showed excellent removal ability, with a cadmium removal rate of up to 95.93%, and that this method had no negative impact on the primary composition and structure of rice. Furthermore, NADES may be effectively separated from rice flour because it is highly soluble in water. It is achievable to lower the amount of NADES in rice noodles to an acceptable level without causing secondary contamination [34]. Yang et al. [35] first studied the elution effect of NADES on Pb, Cd, Cr, As and Cu in *Porphyra haitanensis.* Compared with pure water in the control group, 28 kinds of NADESs significantly increased the removal rate of heavy metals, and they did not affect the sensory quality of *Porphyra haitanensis*. Ochratoxin A (OTA) is a food contaminant, which is great potential threat to human health. Piemontese et al. [57] used NADES to extract OTA from wheat, breadcrumbs and biscuits for the first time. According to the findings, the NADES, which was made of choline chloride and urea, was efficient at extracting and solubilizing OTA, with a recovery rate of up to 89%. Overall, choline chloride-based NADES is commonly used to analyze heavy metals and other pollutants in food. This is because choline chloride is easily biodegradable, reaching 93% degradation in 14 days.

### 4.3. Extractant

The effectiveness of an extractant is attributed to its solubility properties. The extensive hydrogen bonding network within NADES allows it to exhibit excellent solubilization and stabilization ability [58]. In fact, NADES has been reported as an extractant to extract various compounds from food products (Table 1). Most of the studies used ultrasound- and microwave-assisted NADES for extraction. On this basis, researchers further explored the effect of NADES on the stability of the extract. Jelński et al. [59] compared the stability of curcumin in extracts obtained with NADES and methanol, and they showed that NADES could prevent the photodegradation of curcumin during storage. Stability testing of carthamin showed that it was twice as stable in NADES at 60 °C as in water, and five times more stable in NADES at 40 °C than at 60 °C. This is due to the molecular interaction between sugary NADES and compounds to protect the extract from degradation by heat, light and time, thus improving the stability of biological components [60]. Similarly, Liu et al. [61] studied the curcuminoids’ antioxidant activities and stabilities in different solvents. The results showed that curcuminoids were more stable and had better antioxidant activity in NADES composed of organic acids and sugars than in organic solvents.

### 4.4. Taste Enhancer

NADES is used as a green flavor enhancer due to its non-toxicity, low cost and safe consumption. The Maillard reaction is one of the typical reactions that gives food its unique flavor. Kranz et al. [50] explored the effect of NADES on the Maillard reaction for the first time, and studies showed that NADES could indeed improve the formation speed of various Maillard-derived taste regulators. Among them, the sugar-based NADES showed the best promotion, with a yield of 489.0 μmol/mmol of flavor enhancer obtained. Moreover, the effect of NADES on the yield of thiamine-derived taste regulator was studied, and it was observed that NADES composed of cysteine showed a higher productivity than a pure water system. In addition, it was found that the production rate of thiamine-derived taste regulator was higher in alkaline NADES, and increased with increasing NaOH content [62]. Interestingly, the yield of Maillard-derived flavor enhancers by NADES is temperature dependent, while the rate of thiamine-derived flavor enhancers is pH dependent.

### 4.5. Cling Film

NADES is also used in food films, and most studies have focused on the effects of phenolic compounds obtained from food by-products by NADES, on the mechanical and functional properties of food films, and individual researchers have explored the incorporation of NADES into films as a plasticizer. Chitosan is usually used as a cling film to improve food quality, and its film properties are mainly affected by the plasticizer. Galvis-Sánchez et al. [63] studied four choline chlorate-based NADESs as plasticizers for thermoplastic chitosan films, and found that films containing NADES had the best elasticity, the lowest permeability and higher tensile strength values. The presence of NADES will also change the water absorption performance of the film. Composite chitosan films with more NADES have stronger water absorption capacity in high relative humidity environment and better mechanical properties under low water content [64]. In particular, synthetic foods coated with NADES contribute to the elimination of waste produced by traditional outer packaging materials, enhancing the economic value of freshly coated foods while also providing safety, economy and sustainability [65].

## 5. Limitations and Challenges

NADES has the advantages of low cost, simple synthesis, low vapor pressure, good thermal stability and repeatability. It is widely used for food analysis, food films and extracting different compounds. Although great progress has been made in the application of NADES in the food field, there are still some limitations and challenges. Due to the low toxicity of some NADESs, the application of NADES as a solvent is still in laboratory scale, which limits its wide application in food processing. Moreover, NADES usually has a high viscosity, which affects the extraction efficiency. Although ultrasound-assisted NADES extraction is highly efficient, it is limited to small-scale extraction. Coupled with the fact that NADES is non-volatile, the technology to extract bioactive compounds from food by NADES is still limited. In addition, it has been explored how various synthesis approaches affect the properties of NADES. However, the purity of NADES prepared using various methods remains to be solved.

## 6. Conclusions

NADES is mainly composed of natural biological metabolites such as sugars, amino acids, organic acids and choline derivatives, and can be synthesized by six physical methods. It has excellent properties such as biodegradability, antioxidant activity, antibacterial activity, low or non-toxicity and wide polarity range, which are mainly influenced by composition, water content, and temperature. In addition, this review describes the mechanism of action of NADES in the three aspects of cryoprotection, removal of heavy metals from food and extraction of phenolic compounds. Based on this, the applications of NADES in food cryopreservation, food analysis and extraction of active compounds, and as flavor enhancers and food films, are summarized. Overall, as a novel green solvent, NADES shows great potential for application in various aspects of food.

## 7. Future Prospect

In order to promote a broader and more comprehensive application of NADES, this review gives an overview of the latest directions of NADES applications, which include the following four main areas. (i) NADES provides a green alternative to conventional organic solvents. Compared with organic solvents, NADES not only has a high extraction efficiency, but it can also increase an extract’s stability. Green bioactive ingredients can be extracted by NADES and applied to cosmetics, laying the foundation for the green transformation of the cosmetics industry. (ii) Amino-acid-based NADES is safe, non-toxic, widely sourced, low-cost and nutritious, and can be used as a new nutritional ingredient in the food market or as a nutritional additive in the pharmaceutical field. (iii) Because NADES’s characteristics can be adjusted, it provides the possibility to prepare solvents with specific properties and opens up the prospect of creating specialized solvents. (iv) NADES has antifreeze and antibacterial properties, indicating the great potential of NADES as a novel antifreeze agent in food. Moreover, this property of NADES provides a possibility for the development of biomimetic antifreeze materials and opens a new path for novel interfacial antifreeze agents.

## Figures and Tables

**Table 1:** Application of NADES.

Application		Food Matrix	NADES	References
Types	Target Compound			
Cryoprotectant		Chicken breast	Proline: Glucose (1:1), Urea: Glucose: Calcium chloride (3:6:1) Proline: Sorbitol (1:1), Proline: Glucose (5:3),	[32]
Food analysis	Lead and cadmium	Edible oils	Choline chloride: Urea (1:2), Choline chloride: Oxalic acid (1:2) Choline chloride: Ethylene glycol (1:2)	[39]
	Cobalt	Tea samples	Choline chloride: Phenol (1:1, 1:2, 1:3, 1:4)	[40]
	Pesticide residues	Fruit juices and vegetables	Choline chloride: o-Cresol (2:1), Choline chloride: p-Cresol (2:1) Choline chloride: 4-chlorophenol (2:1)	[41]
Extractant	Phenolic compounds	Orange peel waste	Choline chloride: Ethylene Glycol (1:2, 1:3, 1:4) Choline chloride: Glycerol (1:2, 1:3, 1:4)	[42]
		*Carya cathayensis*	Choline chloride-Malic acid, Choline chloride-Lactic acid	[36]
		Agro-food	Lactic acid: Glucose (5:1), Citric acid: Glucose (1:1) Fructose: Citric acid (1:1)	[43]
		*Sapodilla pulp*	Choline chloride: Malic acid (3:2), Choline chloride: Glycerol (1:2) Choline chloride: Lactic acid (1:1), Choline chloride: Urea (1:2)	[44]
		Grain	Choline chloride: Glycerol (1:2)	[45]
	Anthocyanins	Grape pomace	Choline chloride: Citric acid (2:1), Proline: Malic acid (1:1) Betaine: Malic acid (1:1), Betaine: Citric acid (1:1) Choline chloride: Proline: Malic acid(1:1:1)	[46]
		*Myrciaria* *c* *auliflora*	Choline chloride: Propyleneglycol (1:2), Betaine: Citric acid (3:1) Choline chloride: Malic acid (1:1), Citric acid: Glucose (1:1) Choline chloride: Citric acid (1:1)	[47]
Extractant	Anthocyanins	Blackberry	Choline chloride: Acetic acid (1:2), Choline chloride: Urea (1:2) Choline chloride: Glucose (1:2), Choline chloride: Xylitol (1:2) Choline chloride: Lactic acid (1:2), Lactic acid: Sorbitol (1:2) Choline chloride: Citric acid (1:2), Acetic acid: Sorbitol (1:2) Choline chloride: Butanediol (1:2), Malic acid: Xylitol (1:2) Choline chloride: Glycerol (1:2), Citric acid: Xylitol (1:2)	[27]
	Pectin	*Myrciaria* *c* *auliflora*	Choline chloride: Malic acid (1:1), Choline chloride: Citric acid (1:1) Choline chloride: Propyleneglycol (1:2), Betaine: Citric acid (3:1) Citric acid: Glucose: Water (1:1:3)	[47]
	Soluble sugars	Banana	Glucose: Choline chloride (2:3), Fructose: Choline chloride (2:3) Malic acid: Choline chloride (1:1), Malic acid: Fructose (1:1) Malic acid: b-alanine (1:1), Urea: Glucose (1:1) Malic acid: Glucose (1:1), Urea: Fructose (1:1)	[48]
	β-carotene	Pumpkin	Caprylic acid: Capric acid (2:1, 3:1, 4:1), Caprylic acid: Lauric acid (3:1) Pelargonic acid: Lauric acid (3:1), Capric acid: Lauric acid (2:1) DL-menthol: Capric acid (2:1), DL-menthol: Caprylic acid (1:1) Pelargonic acid: Capric acid: Lauric acid (3:1:1)	[49]
Taste enhancer	Novel taste enhancers	Maillard-type taste enhancers	Choline chloride: Sucrose: Water (4:1:4), Glucose: Sucrose: Water (1:1:9) Choline chloride: Urea: Water (1:2:1), Malic acid: Sucrose: Water (1:1:5) Betaine: Sucrose: Water (2:1:9), Betaine: Glycerol: Water (1:2:2)	[50]
Cling film	Bioactive compounds	*Luma chequen* *A. Gray berry*	Lactic acid: Glycerol (1:1, 1:2, 2:1), Choline chloride: Citric acid (5:4) Tartaric acid: Glycerol (1:2, 1:3, 1:4), Choline chloride: Glycerol (4:6) Lactic acid: Glucose (8:1), Glycerol: Glucose (8:1)	[51]

## Data Availability

The data presented in this study are available in the article.
